# Supplementary material for: Fungal Treatment for the Valorization of Technical Soda Lignin
Source: J Fungi (Basel). 2021 Jan 9;7(1):39. doi: 10.3390/jof7010039 (PMC7827817; doi:10.3390/jof7010039)
Supplement: Supplementary file 1 [file jof-07-00039-s001.zip › jof-1060745-fig.pdf]

A.

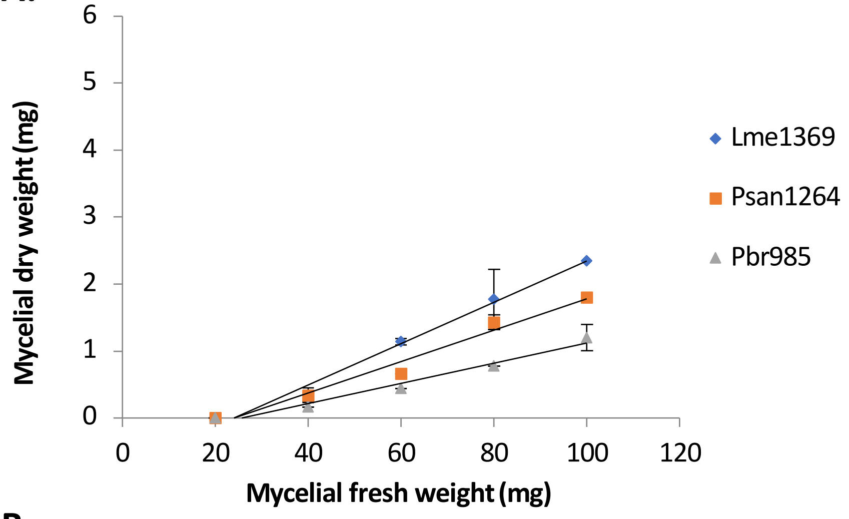

B.

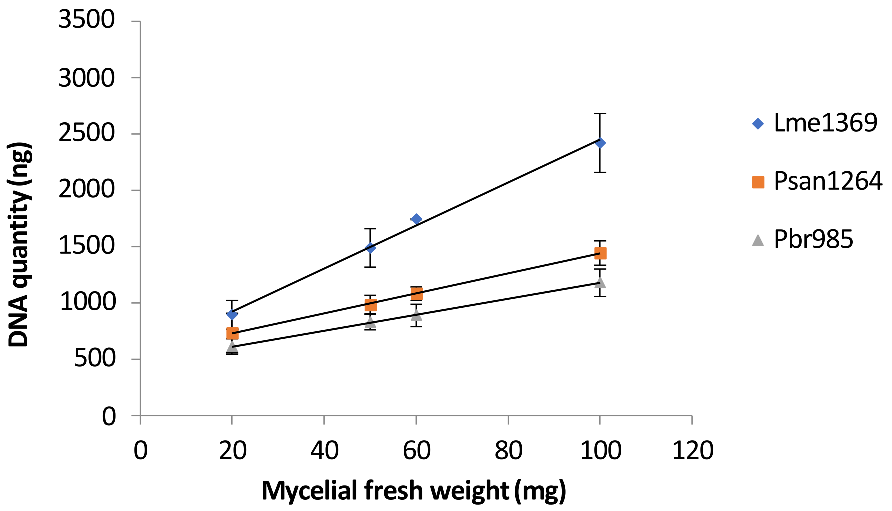

Figure S1. Correlations between mycelial dry weight, mycelial fresh weight, and DNA quantity.

Determination of (**A**) correlation between mycelial fresh weight and dry weight for Lme1369 (f = 0.0308x-0.735; R^2 = 0.9856), Psan1264 (f = 0.0235x-0.561; R^2 = 0.9743), and Pbr985 (f = 0.0151x-0.39; R^2 = 0.9741); (**B**) correlation between DNA quantity and mycelial fresh weight for Lme1369 (f = 19.066x+541.73; R^2 = 0.9957), Psan1264 (f = 8.8786x+551.23; R^2 = 0.9995), and Pbr985 (f = 7.0496x+473.4; R^2 = 0.9996)

LC-MS/MS Q-Exactive Plus analysis

Protein digest was loaded at 7.5 µL/min on a precolumn (C18 particle 5 µm size, 20 mm length, 100 µm i.d., NanoSeparation) and desalted with 0.1 % formic acid in 2 % ACN. After 4 min, the precolumn was connected to a separating column (C18 particle 3 µm size, 300 mm length, 75 µm i.d., NanoSeparation). Buffers were 0.1 % formic acid in water (solvent A) and 0.1 % formic acid in ACN (solvent B). Peptide separation was achieved using a linear gradient from 5 to 35 % of solvent B for 75 min at 300 nL/min totaling 95 min including the regeneration and equilibration steps.

MS data acquisition included a full MS scan covering 350 to 1400 mass-to-charge ratio (m/z) with a resolution of 70000. HCD fragmentation (MS/MS) step was reiterated for the 8 major ions detected during the full MS scan with normalized collision energy of 27 and a resolution of 17500.

LC-MS/MS LTQ-orbitrap analysis

Protein digest was injected and preconcentrated on a precolumn (Acclaim PepMap C18 particle 5 µm size, 5 mm length, 300 µm i.d., Thermo Fisher Scientific) at 20 µL/min with 0.08 % TFA in 2 % ACN in 2 min, followed by a separation on reverse phase separating column (Acclaim PepMap RSLC nanoViper, C18 particle 2 µm size, 150 mm length, 75 µm i.d., Thermo Fisher Scientific). Buffers were 0.1 % formic acid in 98 % water (solvent A) and 0.1 % formic acid in 80 % ACN (solvent B). The peptides were eluted with a multi-step gradient from 1 to 35 % of solvent B for 79 min at 300 nL/min for a total run of 90 min.

MS scans were acquired in a mass range of m/z 300-1400 at a resolution of 15000 in the orbitrap analyser. The 8 most intense ions were selected for CID MS/MS with normalized collision energy of 35 in the ion trap.

Experiment description S*2*. Parameters and conditions for the LC-MS/MS Q-Exactive Plus and LC-MS/MS LTQ-orbitrap analyses.

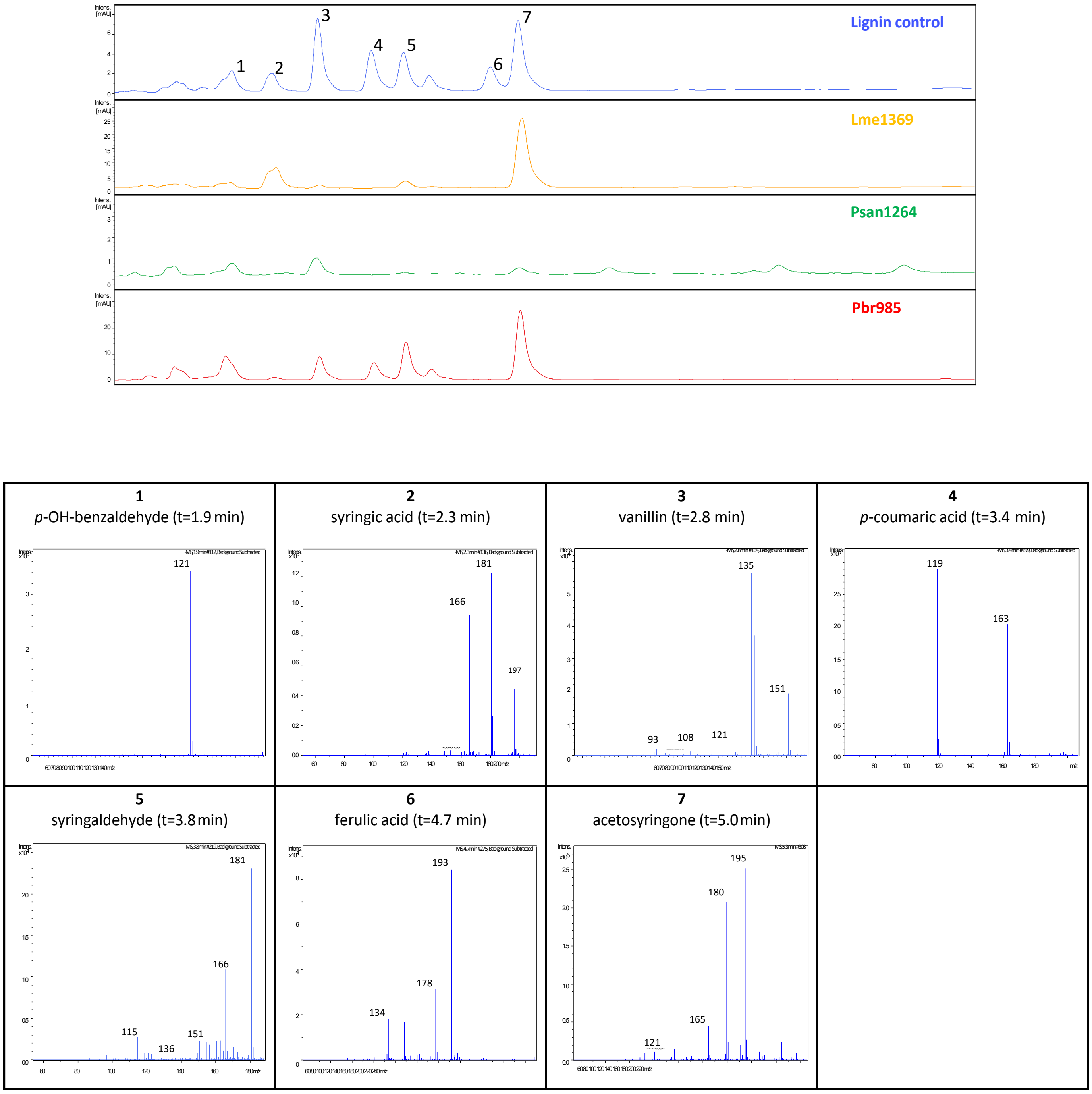

Figure S3. LC-MS analysis on water-soluble lignin fraction. The analysis is done on phenolic monomers extracted from the culture supernatant by ethyl acetate. Normalized chromatograms obtained with a C18 column (Highpurity, Thermo Electron Corporation, 2.7 µm, 50 mm x 2 mm I.D.mm), a 5–100 % vol. aqueous acetonitrile, 1‰ HCOOH gradient (30 min) and 0.4 $ml.min^{-1}$ flow rate, and with a 280 nm UV detection. ESI-MS spectra were obtained in the negative mode from scans acquired in a mass range of m/z 120–2000.

Strain	Protein ID	Signal Peptide
Pbr 985	1421205	No
	1485200	Yes
	1405628	No
	1355486	Yes
	1401242	Yes
	1551444	No
	1483812	Yes
	1362054	Yes
	1348020	No
	1375769	No
	1393086	No
	1410586	No
	1346110	No
	1359728	No
	1408082	No
	1412015	No
	1481969	No
	1392401	No
	1411356	No
	1449227	No
	1399305	No
	1357935	Yes

Strain	Protein ID	Signal Peptide
Psan1 264	1671724	Yes
	1758278	Yes
	1538639	No
	1593127	No
	1593246	No
	1622182	No
	1561162	No
	1573469	No
	1573641	No
	1657735	No
	790266	Yes
	1652042	No
Lme1 369	1115561	No
	984637	Yes
	909456	Yes
	1052862	Yes
	1013677	No
	1006560	Yes
	916898	No
	924929	Yes
	1050535	No
	46074	Yes
	969197	No

Strain	Protein ID	Signal Peptide
	930863	No
	271833	Yes
	926020	No
	1055291	Yes
	1059517	No
	377270	Yes
	971010	No
	974261	No
	909220	No
	1046738	No
	954400	No
	1105316	Yes
	1087844	Yes
	668678	No
	995449	No
	924154	Yes
	326091	Yes
	1058666	No
	1108970	Yes

Table S5. Other proteins identified in the secretomes on lignin alone and their predicted signal peptides.
